# The Evolutionary Paradox of Tooth Wear: Simply Destruction or Inevitable Adaptation?

**DOI:** 10.1371/journal.pone.0062263

**Published:** 2013-04-24

**Authors:** Stefano Benazzi, Huynh Nhu Nguyen, Dieter Schulz, Ian R. Grosse, Giorgio Gruppioni, Jean-Jacques Hublin, Ottmar Kullmer

**Affiliations:** 1 Department of Human Evolution, Max Planck Institute for Evolutionary Anthropology, Leipzig, Germany; 2 Dental Workshop Bensheim, Private Laboratory for Training, Research and Methods, Heppenheim, Germany; 3 Department of Mechanical and Industrial Engineering, University of Massachusetts, Amherst, Massachusetts, United States of America; 4 Department of Cultural Heritage, University of Bologna, Ravenna, Italy; 5 Department of Palaeoanthropology and Messel Research, Senckenberg Research Institute, Frankfurt am Main, Germany; University of Pennsylvania, United States of America

## Abstract

Over the last century, humans from industrialized societies have witnessed a radical increase in some dental diseases. A severe problem concerns the loss of dental materials (enamel and dentine) at the buccal cervical region of the tooth. This “modern-day” pathology, called non-carious cervical lesions (NCCLs), is ubiquitous and worldwide spread, but is very sporadic in modern humans from pre-industrialized societies. Scholars believe that several factors are involved, but the real dynamics behind this pathology are far from being understood. Here we use an engineering approach, finite element analysis (FEA), to suggest that the lack of dental wear, characteristic of industrialized societies, might be a major factor leading to NCCLs. Occlusal loads were applied to high resolution finite element models of lower second premolars (P_2_) to demonstrate that slightly worn P_2_s envisage high tensile stresses in the buccal cervical region, but when worn down artificially in the laboratory the pattern of stress distribution changes and the tensile stresses decrease, matching the results obtained in naturally worn P_2_s. In the modern industrialized world, individuals at advanced ages show very moderate dental wear when compared to past societies, and teeth are exposed to high tensile stresses at the buccal cervical region for decades longer. This is the most likely mechanism explaining enamel loss in the cervical region, and may favor the activity of other disruptive processes such as biocorrosion. Because of the lack of dental abrasion, our masticatory apparatus faces new challenges that can only be understood in an evolutionary perspective.

## Introduction

**Figure 1:**
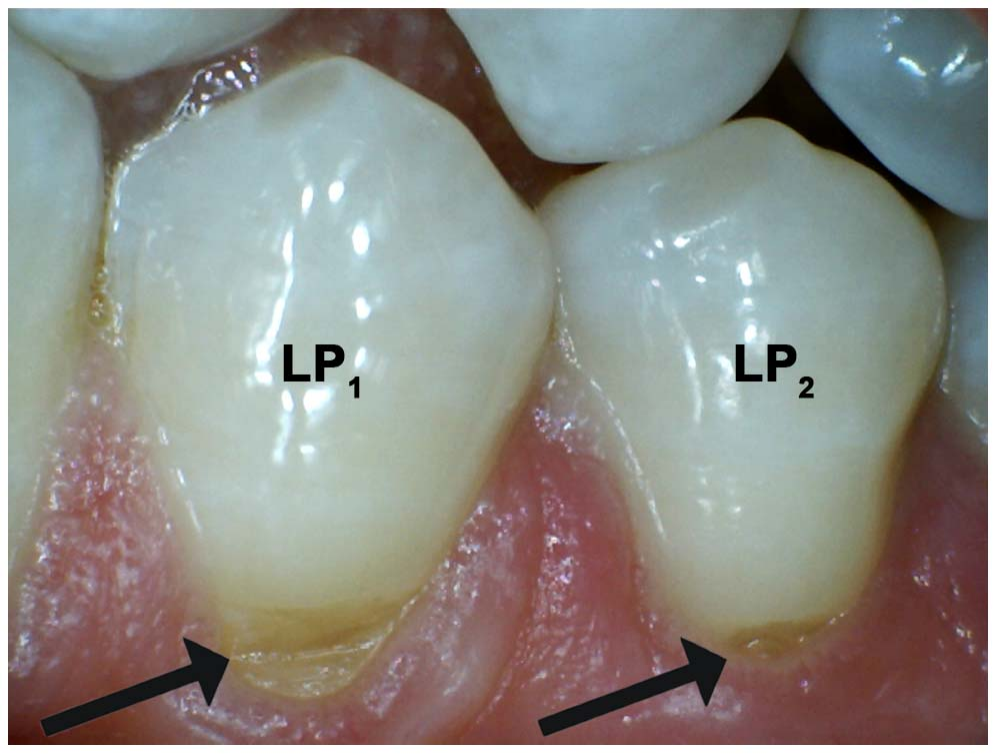
Modern lower premolars presenting non-carious cervical lesions (NCCLs). Arrows point towards the NCCLs in the buccal cervical region of the lower left first and second premolar (LP_1_ and LP_2_, respectively).

Wedge-shaped defects in the buccal cervical region of the tooth, known as non-carious cervical lesions (NCCLs) (Figure 1) [1]–[3], do not find any parallel in the ancestral human lineage and are very sporadic in modern humans from pre-industrialized societies [4]–[6]. To account for the worldwide spread of NCCLs scholars have advocated multifactorial aetiologies, such as toothbrush/dentifrice abrasion, biocorrosion, and abfraction. Abrasion is considered a physical mechanism causing wear by friction of physical–chemical agents on the tooth surface [7]–[9]. Biocorrosion represents the chemical, biochemical and electrochemical degradation of dental tissues due to endogenous and exogenous acids, by biochemical proteolytic enzymes and piezoelectric effects [3], [10], [11]. The term abfraction was introduced by Grippo [14] to underline the loss of hard tissue in the cervical region of the tooth by non-axial forces exerted on the occlusal surface, which cause microfractures of dental tissues in areas of stress concentration [3], [12], [13]. For a review of the purported causes for NCCLs, the reader may refer to the copious literature on this topic (i.e., [1], [3], [15], [16]).

Scholars disagree about the relative importance of these factors. NCCLs have been observed in modern populations wherein toothbrush and dentifrice were not in use [17] and biocorrosion alone cannot satisfactorily explain this phenomenon. Indeed, lesions have been observed in subjects with no acidic raw food intake [18] and they can only affect a single tooth [15]. Wedge-shaped defects are frequently located subgingivally, which argue against both toothbrushing abrasion and biocorrosion as being the main contributing factors [18], [19]. Moreover, to our knowledge, NCCLs have not been observed in deciduous teeth, further undermining toothbrushing and biocorrosion as the main causes. The role of abfraction has also been widely challenged, as it is controversial and poorly understood [15], [16]. To support the role of occlusal forces in the development of NCCLs in the buccal cervical region of the tooth, scholars have mostly used finite element analysis (FEA) [12], [13]. However, the some approach has also provided contradictory evidences, given that tensile stresses were observed also in the lingual cervical region of the tooth, an area where NCCLs are rarely observed [20]. Scholars have also searched for a correspondence between NCCLs and malocclusion [12], as well as between NCCLs and occlusal wear [9], [19], [21], arguing that malocclusion (i.e., angle class II and III) should produce wear facets and that the more wear facets the tooth has, the more likely the tooth is affected by NCCLs (see [22] for a systematic review of the correspondence between NCCLs and occlusal wear). However, evidence goes against this assumption, because in some circumstances no association has been observed between angles classification and NCCLs [23], and because slightly worn teeth (namely, teeth with few wear facets) show NCCLs too [1], [24].

Many erroneous considerations among dental practitioners rely on a fundamental misunderstanding of the concept of tooth wear [2], [22], [25], seen more as the result of malocclusion or atypical occlusal loading than as a natural physiological process. Extensive tooth wear was ubiquitous in every past-populations of the world, who consumed less refined and processed foods [25], [26]. However, in the last century people of most modern societies have experienced a dramatic decrease in dental wear due to the consumption of softer and cleaned food items and differences in lifestyle [25], [26]. As suggested by Kaifu and colleagues [26], the discrepancy between “the original design of our dentition and our present environment” might explain the increase in frequency of some dental diseases in contemporary societies. For example, mesial drift, continuous eruption, lingual tipping of the anterior teeth might be evolved as compensatory mechanisms for heavy interproximal and occlusal wear. Alterations of these compensatory mechanisms can be, for example, important factors leading to malocclusion [26]. Moreover, Aubry and colleagues [4] concluded that the lack of wear characteristic in modern humans of contemporary societies may play a major role in the development of NCCLs.

In this contribution we describe an investigation of Aubry and colleagues [4] hypothesis about the relation between the lack of tooth wear and the risk of NCCLs. Testing this hypothesis through biomechanical *in vivo* experiments is impossible due to practical and ethical reasons. *In vitro* biomechanical tests of complete teeth would be difficult to perform and inaccurate, due to the small size of the occlusal contact areas (wherein forces should be applied) in comparison to the relative large size (for a tooth) of available strain gauges. Therefore, we tested the effects of tooth wear using three-dimensional (3D) FEA [27]. While in previous contributions based on 3D FEA less attention was devoted to the loading conditions, as most of the scholars simplify forces to point loads (i.e., [12], [13], [28], [29]), here we apply a newly developed advanced loading concept derived from the individual occlusal wear information [30], [31]. Lower premolars are often affected by NCCLs. We therefore compared maximum principal stresses in slightly worn (specimen S23 and S81) and heavily worn (specimen S5 and S126) lower right second premolars (RP_2_s) during maximum intercuspation contact (tooth-to-tooth contact; load = 100 N). Afterwards, specimen S23 and specimen S81 were artificially worn down (hereafter referred as S23w and S81w, respectively) to directly evaluate the effects of wear on the stress distributions.

## Results

**Figure 2:**
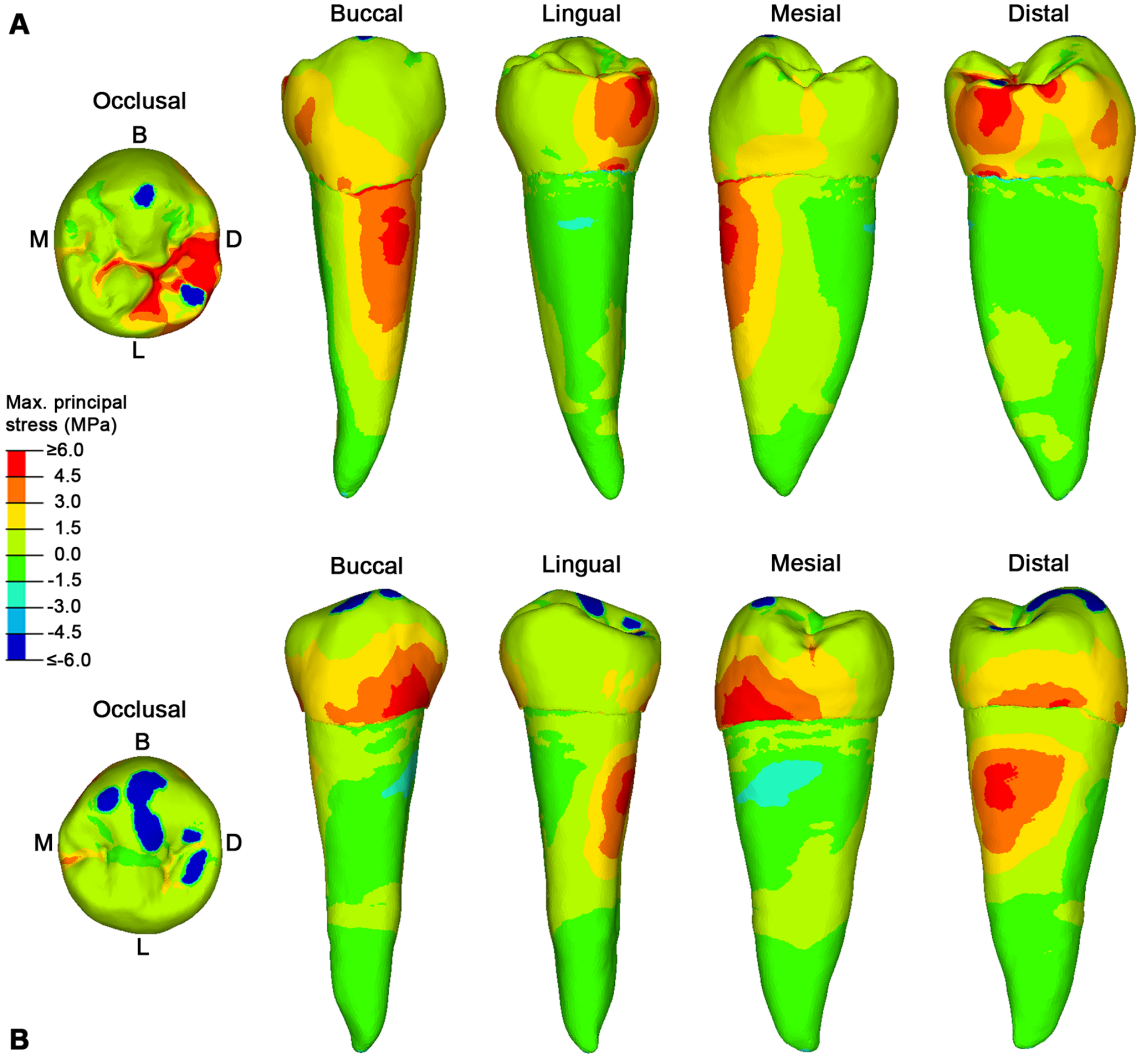
The maximum principal stress distribution for specimen S23 and S81 lower right second premolars (RP_2_). A, specimen S23 in occlusal, buccal, lingual, mesial and distal view. B. specimen S81 in occlusal, buccal, lingual, mesial and distal view. Blue spots in the occlusal surface (compressive stress) represent the contact areas with the antagonistic teeth, during maximum intercuspation (see Video S1 for specimen S23, and Video S2 for specimen S81), where the load was applied. Red spots represent tensile stresses. B, buccal; D, distal; L, lingual; M, mesial.

During maximum intercuspation, slightly worn RP_2_s present contact areas mainly localized on the buccal cusp and in the distal margin of the occlusal surface (Figure 2A,B). Due to the steepness of the buccal cusp, the load is directed obliquely with respect to the axis of the tooth. Consequently, the buccal side of the tooth experiences high tensile stresses, affecting the cervical half of the root in S23 (Figure 2A) and the lower third of the crown in S81 (Figure 2B). Differences in the pattern of stress distribution between the two specimens is due to differences in morphology of the teeth and to the different wear stages of the two specimens, as S81 is more worn than S23. Moreover, the high tensile stresses observed in the roots of S23 (buccally; Figure 2A) and S81 (distally; Figure 2B) might also account for another major clinical issue currently faced by dental clinicians, namely root fracture.

**Figure 3:**
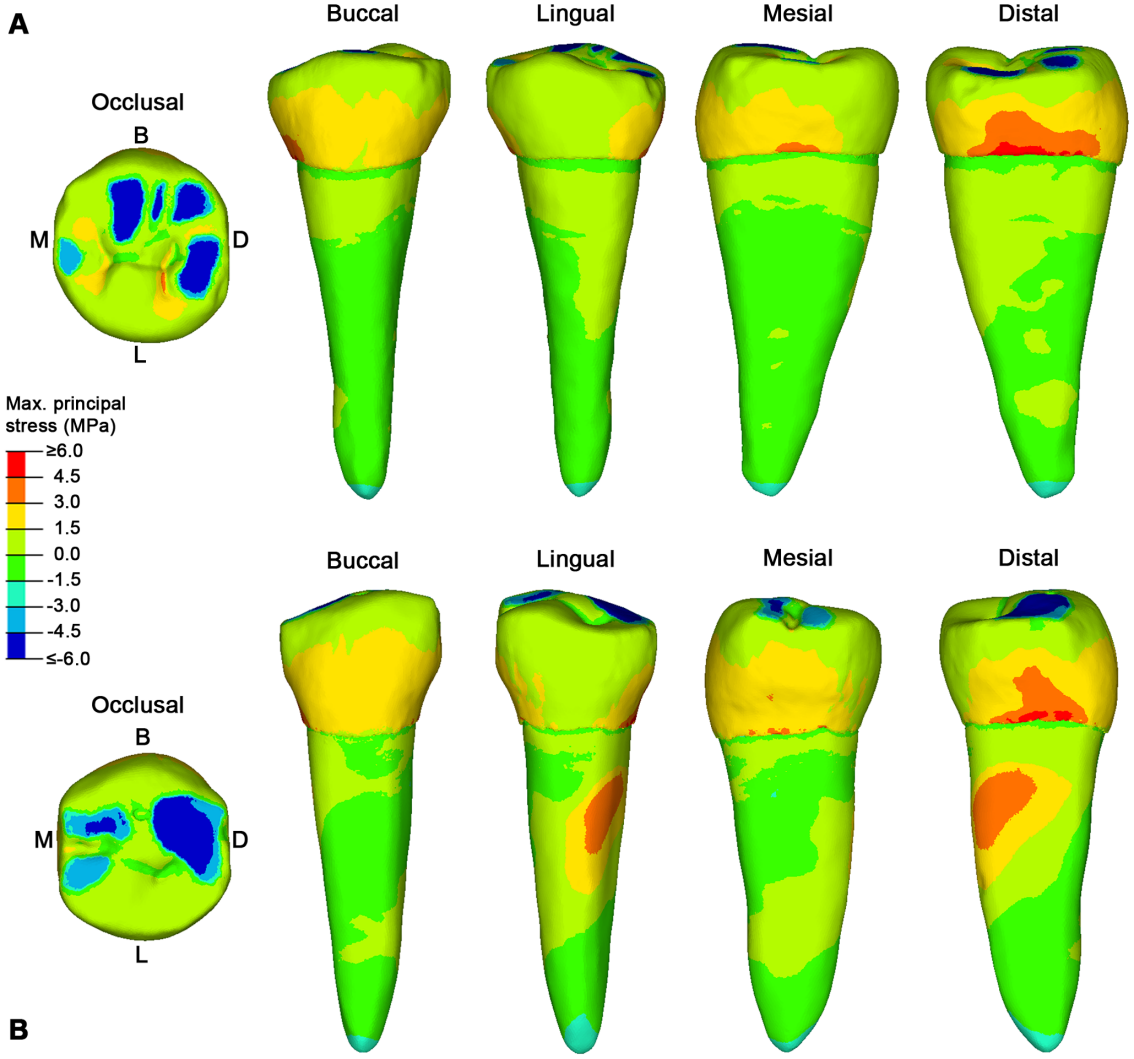
The maximum principal stress distribution for specimen S5 and S126 lower right second premolars (RP_2_). A, specimen S5 in occlusal, buccal, lingual, mesial and distal view. B. specimen S126 in occlusal, buccal, lingual, mesial and distal view. Blue spots in the occlusal surface (compressive stress) represent the contact areas with the antagonistic teeth, during maximum intercuspation (see Video S3 for specimen S5, and Video S4 for specimen S126), where the load was applied. Red spots represent tensile stresses. B, buccal; D, distal; L, lingual; M, mesial.

In worn teeth (S5 and S126), contact areas cover a larger portion of the occlusal surface, favouring a less localized distribution of the load. Since the occlusal reliefs are reduced, the occlusal load is almost parallel to the longitudinal axis of the tooth and the force is directed towards the root’s apex, which indeed shows compressive stresses (Figure 3A,B). Due to the decrease of the non-axial loadings (i.e. bending loads), the tensile stresses are reduced and affect the lateral sides of the teeth, mainly distally, instead of the buccal region. Interestingly, the lack of tensile stresses in the lingual side is in agreement with only a sporadic presence of NCCLs in this region of the teeth.

**Figure 4:**
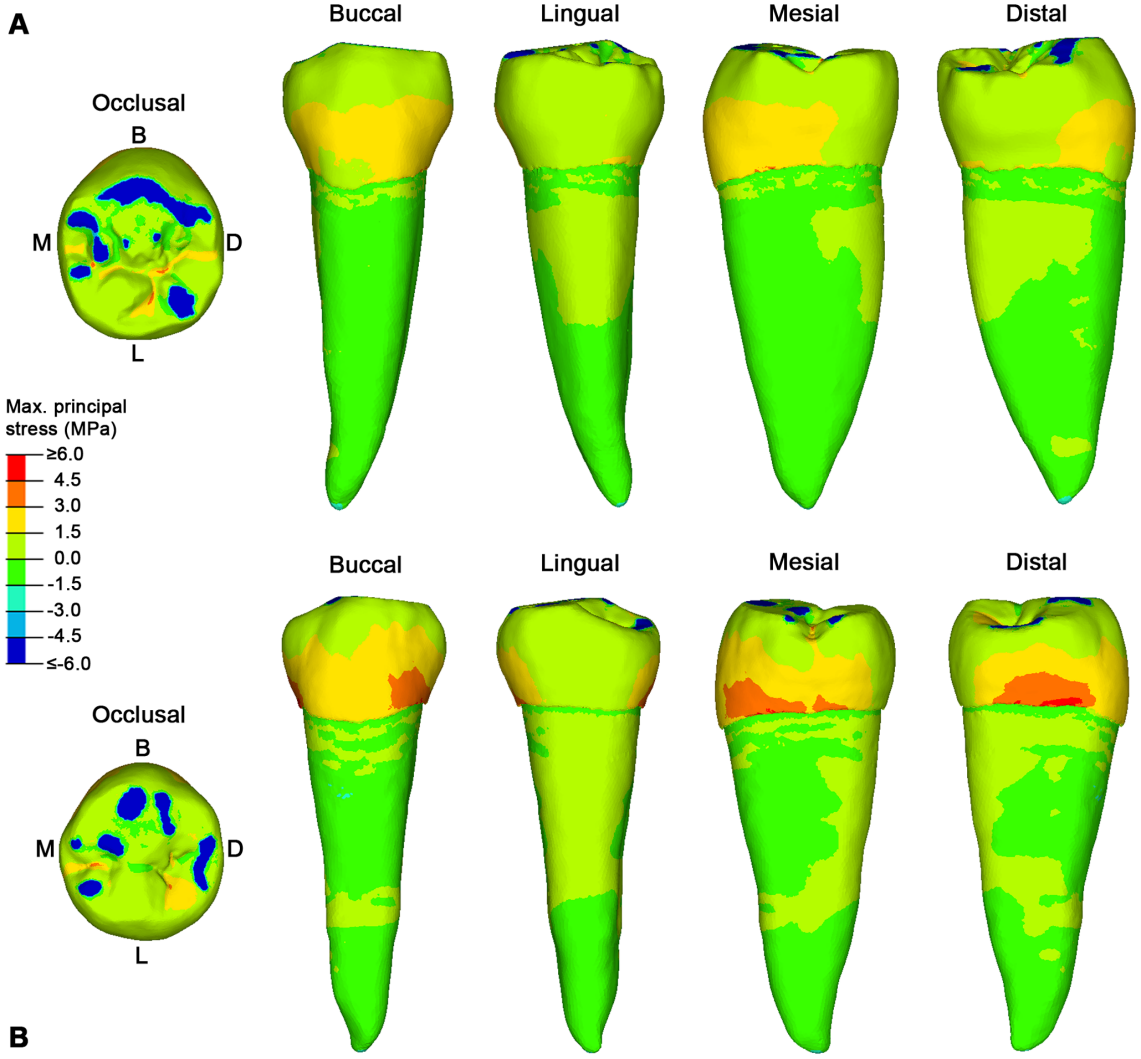
The maximum principal stress distribution for specimen S23w and S81w lower right second premolars (RP_2_). A, specimen S23w in occlusal, buccal, lingual, mesial and distal view. B. specimen S81w in occlusal, buccal, lingual, mesial and distal view. Blue spots in the occlusal surface (compressive stress) represent the contact areas with the antagonistic teeth, during maximum intercuspation (see Video S5 for specimen S23w, and Video S6 for specimen S81w), where the load was applied. Red spots represent tensile stresses. B, buccal; D, distal; L, lingual; M, mesial.

**Figure 5:**
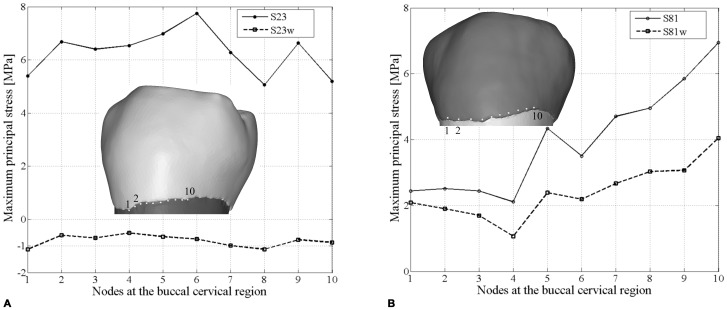
Differences in tensile stress values between the original (S23 and S81) and the artificially worn down (S23w and S81w) lower right second premolars. A, the maximum principal stress values for specimen S23 and S23w based on 10 homologous nodes in the buccal cervical region. B, the maximum principal stress values for specimen S81 and S81w based on 10 homologous nodes in the buccal cervical region.

When specimen S23 and S81 are artificially worn down (S23w and S81w, respectively), the contact areas increased in number and extension, the pattern of stress distribution changes accordingly and the tensile stresses in the teeth decrease meaningfully, particularly in the buccal side (Figures 4A,B). Indeed, as shown in the plots of Figure 5A and 5B, tensile stress values in the buccal cervical region are notably higher in the original (S23 and S81) than in the artificially worn down (S23w and S81w) RP_2_s. While in specimen S23w tensile stresses are generally low and mainly concentrated in the grooves of the occlusal surface, in specimen S81w tensile stresses interest the sides of the tooth, as previously observed in the worn specimens S5 and S126. Accordingly, this result shows that tooth wear changes the stress distribution, independently on the primary morphology of the tooth.

## Discussion

Dental wear is a physiological process that progressively affects mammalian teeth throughout their functional life. Being a ubiquitous phenomenon, with obvious positive correlation with the individual age, we can assume that dental wear has played an important role in mammalian dental evolution, where the progression of volume loss of dental materials must have been somehow calibrated depending on the tooth architecture and the lifetime of each species. In fact, it has been suggested that the morphology of the crown might have been selected to maintain chewing efficiency throughout the life of the individual as the tooth wear increases [32]. The topography of the occlusal surface, albeit functionally important for food processing, does not represent the tooth as a whole. In order to explain the functional biomechanics of a tooth both its external (i.e., crown and root shape) and internal architecture (i.e., dentine volume, enamel thickness and its microstructural organization) have to be considered in response to their occlusal load. We can assume that the dental architecture is designed to withstand and distribute the high stress produced during masticatory loadings to their supporting structures. In this regard, since tooth wear changes both the loading position and the loading direction of the occlusal forces [30] and hence changes the distribution of stress (mainly tensile stresses) on the whole tooth. The wear process might have had a crucial influence in the evolution of teeth able to reduce high tensile stresses at the cervical margin in the advanced period of an individual’s lifetime.

To test this hypothesis it was important to overcome limits of current FEA studies, which simplify occlusal forces to a point load, e.g. placed in the central basin of the tooth crown or on the cusp tips (i.e., [12], [13], [28], [29]). We have considered individual tooth-tooth contact scenarios, which might be more damaging to the tooth crown than food-tooth contacts, because more localized stresses increase [33]. Based on advanced loading concepts derived from individual occlusal wear information (see methods below), for the first time the pattern of stress distribution in teeth (namely, lower second premolars) can be evaluated in different wear stages derived from the physiological crown contacts. Additionally, the experimental individual wear simulation enabled us to directly assess the potential effects of advanced tooth wear on the same specimen (Figures 4A,B, 5A,B).

Our results confirm that the occlusal loads are transferred to the crown following a direction dictated by cusp inclination, lending support to the hypothesis that abfraction is a main factor involved in NCCLs [12], [13]. Since in unworn to moderately worn RP_2_s occlusal contact areas are mainly localized in the steeply inclined slopes of the buccal cusp, the occlusal force will be fragmented in lateral loadings that ultimately create tensile stresses in the buccal cervical region of the tooth. Moreover, the concentration of tensile stresses in the root maybe also account for another worldwide dental failure, root fracture [34]. Root fracture is frequently encountered emergency in a dental clinic, but to our knowledge it is not a matter of concern in paleopathological researches of jaw remains. However, our findings should be considered preliminary and need to be confirmed in further studies and enlarged samples.

Aubry and colleagues [4] suggested that as the buccal cusp becomes flatter and the contact areas with the antagonistic teeth increase, the occlusal stresses should decrease, reducing the risk of NCCLs. Indeed, our results confirm this assumption. In our worn specimens (both the original and the experimentally worn teeth), the load directions change from oblique to nearly parallel direction to the dental axis, and locally directed stresses ultimately reduce, thus improving the dispersion of occlusal forces. Therefore, the morphological modification of the occlusal surface resulting from wearing seems important to balance the pattern of stress distribution to which a tooth is subjected during the lifespan of an individual, reducing and shifting the tensile stresses from the buccal side (in unworn-slightly worn teeth) to the mesial/distal sides (in teeth with advanced wear) (Figures 2, 3, 4). The biomechanical implications of this shifting of tensile stresses in the lateral sides of the tooth are currently unclear, and further works are warranted.

Modern human populations of pre-contemporary societies were subjected to high rates of tooth wear, due to the abrasiveness of the diet and adherent grit derived from less refined and processed foods (relatively to modern processed soft foods in contemporary societies) [35]–[37]. In such conditions, cusps became progressively lower and flatter already in young adults, and the load direction during mastication changed from oblique to parallel to the dental axis. It is worthwhile to note that this rapidly progressing tooth wear was usually accompanied by a relatively short lifetime of the individuals (when compared with contemporary societies), as the average life expectancy at birth in prehistoric populations (both hunter-gatherers and agriculturalists) has been estimated between 18–25 years, remaining stable or maybe increasing up to 30–35 years in the Middle Ages and 40–45 years in the 19th century [38], [39].

This trend has completely reversed in the last century, due to the fact that a decrease in tooth wear is now associated with an increase in life expectancy, which is currently about 80 years in industrialized countries [39]. While in pre-contemporary societies dentine exposure in premolars is ubiquitous from about the age of 20/25 [35], in the majority of the modern population tooth wear is often limited to enamel in the entire life of an individual. Wearing affects the dentine to a relatively small degree and, in any case, it occurs at a very advanced age [40]. This implies that in contemporary societies occlusal reliefs do not noticeably reduce, wear facets increase only modestly in surface area, and teeth are exposed to high tensile stresses in the buccal region of the teeth for a much longer time due to non-axial loadings. For this reason, some dental practitioners have suggested reducing cusp height relief to decrease tensile stress values [41].

Results from fracture mechanics show that during the loading of teeth enamel cracks usually develop due to tensile stress and propagate depending on the stress distribution. They follow the pathways of tufts (hypocalcified, protein dense fissures) from the internal enamel-dentin junction (EDJ) towards the outer enamel surface (OES) and from the cervix (enamel-cementum junction) towards the occlusal surface [42], [43]. Cracks can grow incrementally, heal, and grow again over a lifetime, providing information about the load history in a tooth [43]. The travel distance of a crack is limited, depending on the load magnitude and enamel thickness. If a crack has reached the OES, it may cause enamel/dentine failure. The magnitude of load alone cannot be considered the only factor involved in the aetiology of NCCLs. Past modern humans had a stronger masticatory apparatus (as suggested by muscle insertions in the cranium and mandible) than contemporary populations and yet in these early modern humans NCCLs have been rarely observed [4], [5]. However, continuous cyclic occlusal loading can lead to the accumulation of cracks, promoting fatigue and maybe ending in delaminating enamel from dentin [42]. Extensive cyclic tensile stresses along the thin enamelled buccal cervical margin favours the occurrence of multiple cracks on the OES, increasing the surface for activity of additional disruptive processes such as biocorrosion (cyclic fatigue stress biocorrosion) [3]. More studies are needed to explore the relation between marginal cracks and NCCLs.

It is important to raise further observations and comments on some limits of our analysis that should be addressed in future works. First, we have considered only six specimens due to the efforts required to develop the FE models and contact areas. Even though we do believe our sample is morphologically representative, more studies are needed to confirm our preliminary results, including other tooth classes across the dental arch, accurate evaluations of *in vivo* condition of the same tooth used in the simulation, and *in* *vitro* experiments. Second, we have attributed isotropic property to the enamel due to practicability of the FEA, but enamel should be considered anisotropic, having different physical properties in different directions and crown regions [44]. Third, the artificially worn dental casts used in the present study (see methods below) obviously simplify the naturally worn condition. Finally, Bondioli and colleagues have recently observed NCCLs in the Neolithic dental sample, including teeth with advanced wear from Mehrgarh, Pakistan [6]. Even though the frequency of the cases observed in the Mehrgarh prehistoric sample is still low (10 individuals out of 225; 4.4%) compared to the currently worldwide diffusion of the pathology (in the range of 5–85% [45]), it nonetheless suggests that other factors, i.e. parafunctional habits, cannot be excluded in the aetiology of NCCLs. However, despite non-axial occlusal loadings might also depend on parafunctional habits (i.e., bruxism [46]), the surprising spread of the disease in contemporary societies must depend on more generalized changes that took place in the last century.

To summarize, our results support Aubry and colleagues’ abfraction hypothesis for the diffusion of NCCLs in contemporary populations [4], suggesting that the lack of tooth wear increases tensile stresses near the buccal cervix of the tooth, augmenting the risk of NCCLs. This main factor might work in concert with additional disruptive processes (i.e., toothbrush/dentifrice abrasion, biocorrosion), which might explain the variability in the appearance of NCCLs shape and surface roughness, and also the tendency for a higher prevalence with increasing age [1], [11]. Moreover, if future studies confirm that alterations of the compensatory mechanisms for heavy tooth wear (such as mesial drift, continuous eruption, lingual tipping of the anterior teeth) are, to some extent, responsible for malocclusion and other dental diseases in contemporary societies [26], the consequences related to the lack of tooth wear might be more serious than generally thought.

In modern societies, the use of our dentition and its pattern of decay have changed dramatically. An unworn dentition is most desired for aesthetic reasons and as a sign of good oral health. For the prevention of NCCLs and maybe even other dental failure (i.e., root fracture), the dental academic community should be amenable to consider physiological tooth wear in an evolutionary perspective to understand its specific role in the dynamics and function of the masticatory system, instead of seeing it as a phenomenon which just acts to the detriment of oral conditions. Ironically, it seems the lack of physiological wear may in fact lead to pathological conditions!

## Materials and Methods

### Sample

We obtained permission from the Department of Anthropology, University of Vienna, to select four dried modern human skulls from the archaeological skeletal sample collected by Rudolf Poech in South Africa in 1907–1909 [47]. The age at death, and when possible the sex, was assessed by the examination of the cranial and postcranial characters [48]–[50]. The first two specimens (ID = S23 and ID = S81, respectively) are young individuals (15–20 years old), while the second two specimens (ID = S5 and ID = S126, respectively) are adults (about 30 years old). The sex was only assessed for specimen S23 (female) and specimen S5 (male).

The four specimens were selected both because of their complete dentition and because their lower right second premolar (RP_2_) differing in wear stage (after Smith [51]): specimen S23 and S81 show wear stage 1 (wear facets are visible (S81) or slightly visible (S23) on the occlusal surface, but they do not coalesce together and there is not dentine exposed), while specimen S5 and S126 show wear stage 2 (wear facets coalesce together) and wear stage 3 (dentine exposed in the protoconid cusp), respectively.

### Micro-CT Scan, Segmentation and 3D Reconstruction

Scanning of the skulls with upper and lower dentition in maximum intercuspation contact was carried out at the Vienna Micro-CT Lab, Department of Anthropology, University of Vienna, with a Viscom X8060 µCT scanner using the following scan parameters: 130 kV, 100 µA, 1.0mm copper filter, 3197×2239 matrix, and 1440 steps during 360° of rotation. Volume data were reconstructed using isometric voxels of`55 µm. The 3D digital surfaces for the lower right premolars and first molar (RP_1_-RM_1_) and the upper right premolars (RP^1^-RP^2^) were obtained in Avizo 7 software (Visualization Sciences Group Inc.).

For the RP_2_ (used for FEA) a complete segmentation of the dental tissues (enamel, root and pulp chamber) and the supporting dental tissues (periodontal ligament - PDL, trabecular and cortical bone) was carried out (Figure S1A,B). To reduce the size of the digital models for FEA, we cut the mandibles distally to the socket of the lower first premolar and mesially to the socket of the lower first molar. Consequently, we considered only the bone tissues surrounding the RP_2_. For RP_1_, RM_1_ and RP^1^-RP^2^, which were used to assess the occlusal contacts with RP_2_ (two-body interactions), only the external surface of the teeth was segmented. The final refinement of the digital models was carried out in Rapidform XOR2 (INUS Technology, Inc., Seoul, Korea). Besides cleaning processes and corrections of defects to create fully closed surfaces, the digital models were optimized for downstream Computer-Aided Engineering (CAE) applications.

### Simulation of Tooth Wear

For the simulation of tooth wear for specimens S23 and S81 we followed indications provided by Kullmer and colleagues [31]. As a first step, there was need to perform a slight repositioning of the crowns to match perfectly the individual occlusal pattern between the antagonists before the experiment in the dental articulator. Therefore we have moulded the original dentition using 3 M ESPE Imprint (TradeMark) II Garant (TradeMark) Light Body (Vinyl Polysiloxane Impression Material). Casts of the crowns were reproduced with dental stone material (hydro-base®, Dentona AG). The dental stone casts were cut into isolated crowns for the premolars and molars and along the midsagittal line for the incisors and the canines. After that all crowns were mounted with dental wax in a best-fit occlusal situation on ARUNDO-FLEX 2000 duett-plates (Baumann Dental GmbH) such as they are used in dental laboratories. The best occlusal fitting was found regarding the wear facet information incorporated in the crown morphology. The restored dental arches of S23 and S81 were setup in a dental articulator (PROTAR, KaVo Dental GmbH) using geometry details (distances edge length of triangle between midcondyle points and incisor point, distances edge length of triangle between the M_2_ metaconid cusp tips and incisor point, angle between both triangles) from the original jaws of the specimens (Figure S2A,B). The attrition of the premolars was carried out following the description in Kullmer et al. [31]. The condyle boxes of the articulator were setup using the information of the individual occlusal movements, which were extracted from moving the specimen in occlusion in all possible directions starting from maximum intercuspation during the repositioning of the crowns, while the condyle boxes are open with no constraints. Accordingly, both specimens (S23 and S81) got their individual setup in the dental articulator (for more information about the setup of the dental articulator see also [52], [53]).

**Figure 6:**
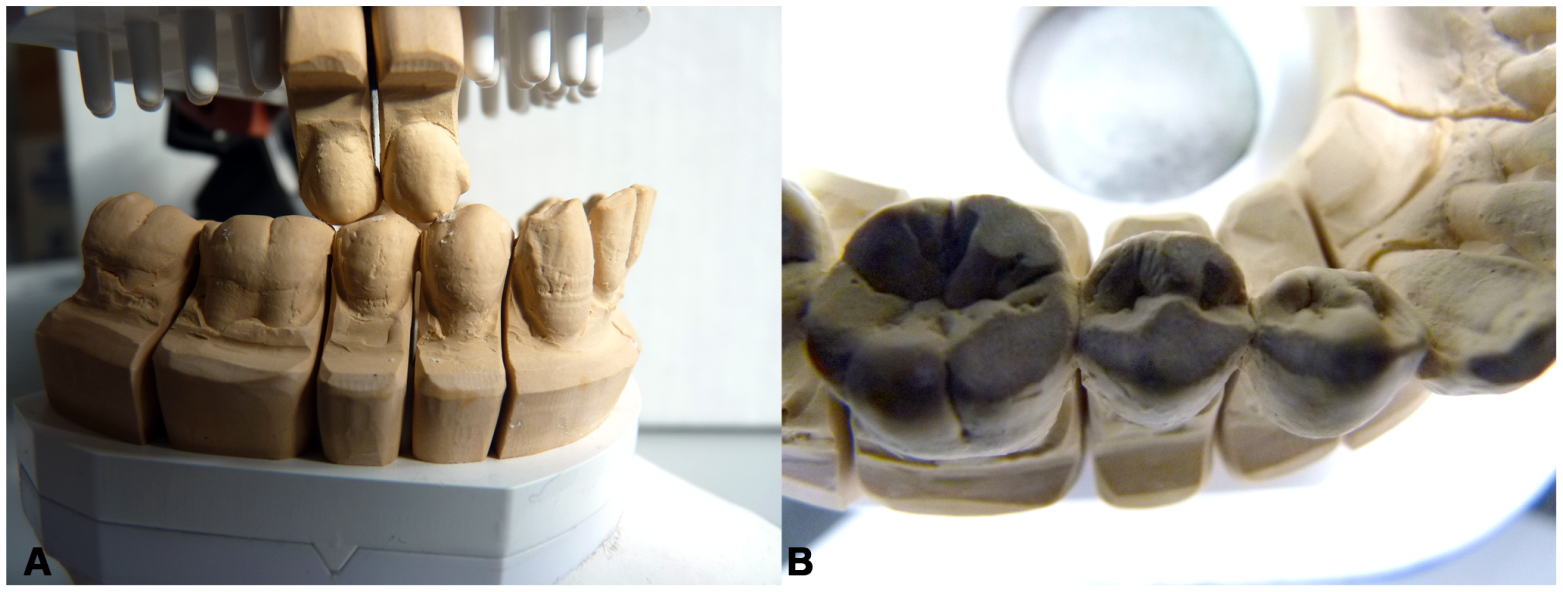
Cast of specimen S81 in the dental articulator (PROTAR, KaVo Dental GmbH). A, buccal view of the specimen during the artificial attrition experiment of the RP_2_ based on the individual pattern of occlusal movements. B, occlusal view of specimen S81w RP_1_-RM_1_ crowns with artificially enlarged wear facets.

After the individual condyle constraints were set, most of the crowns in the upper arch were removed to reduce the occlusal contacts to the region of interest (RP^1^ and RP^2^; RP_1_, RP_2_ and RM_1_) (Figure 6A). This procedure supported a rapid attrition on the RP_2_ grinding the upper against the lower crowns.

The movable upper arm of the fully adjustable dental articulator was moved in occlusion following the directions given through the individual setup. The movements of the antagonists towards each other produced considerable attrition on the dental stone casts (Figure 6B), expressed in the change of crown morphology and extension of occlusal contacts. The artificial tooth wear was stopped when the protoconid tip was oblate, showing distinctive enlarged and flattened wear facets for a new FEA in the worn S23 and S81 RP_2_s (S23w and S81w, respectively).

Finally, the isolated split-cast segments of RP^1^-RP^2^ and RP_1_-RM_1_ crowns were removed and surface scanned using the optical topometry system SMARTSCAN (Breuckmann GmbH) with a resolution of 55 µm [54]. Polygonal surface models were generated using OPTOCAT (Breuckmann GmbH). The complete dental arches in maximum intercuspation were also surface scanned to reference the orientation of the digital RP^1^-RP^2^ and RP_1_-RM_1_ crowns to the articulator’s orientation.

### Loading Position

**Figure 7:**
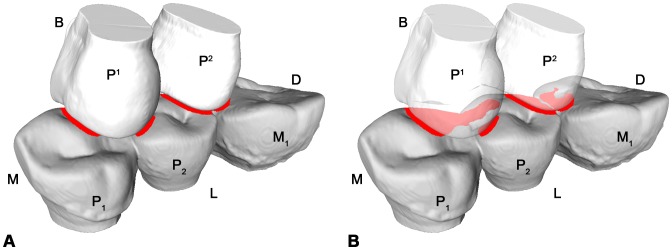
Collision detection for specimen S126 in the Occlusal Fingerprint Analyser (OFA) software. A, mesiolingual view during maximum intercuspation between the lower right premolars and first molar (RP_1_-RM_1_) and the upper right premolars (RP^1^-RP^2^). B, the RP^1^-RP^2^ are transparent to better show the collision (red areas) on the occlusal surface of the RP_2_. See also Video S4. B = buccal; D = distal; L = lingual; M = mesial.

In order to recognize the contact areas on the RP_2_ during maximum intercuspation contact with the antagonistic teeth, the dental surface models of RP_1_-RM_1_ and RP^1^-RP^2^ of the six specimens (S5, S23, S81, S126, S23w and S81w, respectively) were imported into the Occlusal Fingerprint Analyser (OFA) software. The software allows moving one model towards the antagonists along a defined pathway in order to analyse the collision of crown contacts. OFA software prevents the penetration of the models into one another and detects the occlusal contacts through collision detection, deflection and break free algorithms. The colliding triangles of the models are automatically selected by the software and highlighted in a user-defined colour (Figure 7A,B; Video S1–S6).

**Figure 8:**
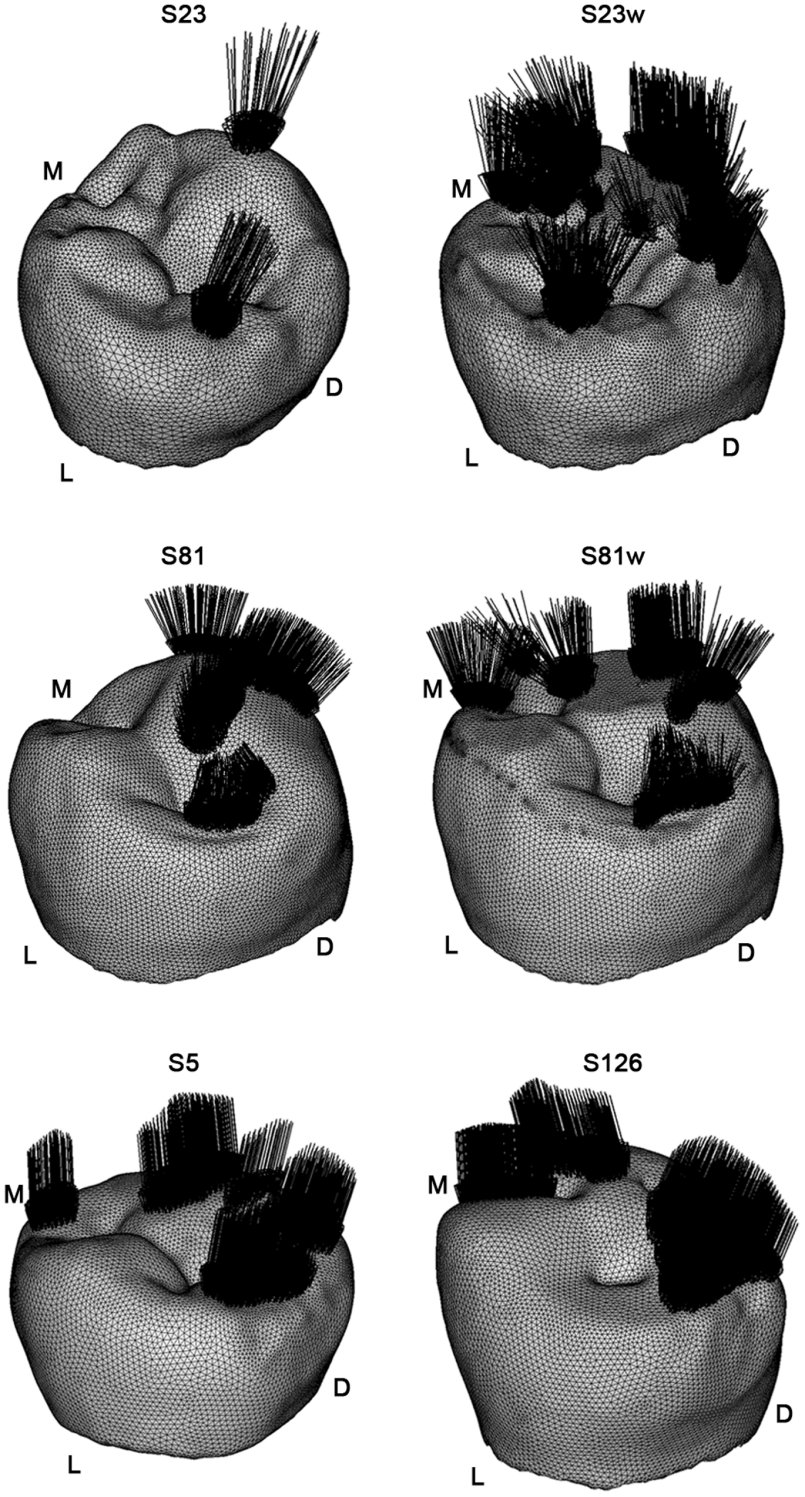
Loading position and direction for specimen S23, S23w, S81, S81w, S5 and S126. For each lower right second premolar (RP_2_) only the volumetric mesh of the enamel is displayed. The load (black arrows) was distributed proportionally according to the occlusal contact areas detected in the Occlusal Fingerprint Analyser (OFA) software. D = distal; L = lingual; M = mesial.

With regard to the loading direction, for maximum intercuspation contact we can assume that a compressive force acts between complementary wear facet pairs, which could ultimately be represented as perpendicular loads to these facets [30], [55]–[56] (Figure 8).

### Finite Element Mesh Feneration and FEA

The surface models were then imported into HyperWorks Software (Altair Engineering, Inc.), where volumetric meshes (for enamel, dentine, pulp, PDL, cortical and trabecular bone shown in Figure S1C) were created using 10-nodes tetrahedral elements (Table S1). For specimen S23w and S81w, the same volumetric meshes of specimen S23 and S81 were used, except for the enamel and the dentine. To include the new information of the artificially worn occlusal surfaces, the RP_2_ digital crowns of S23w and S81w were superimposed to the original RP_2_ crowns in Rapidform XOR2, constraining the superimposition to the regions of the crown unaffected by the wearing. Then, the original RP_2_ occlusal surfaces of specimen S23 and S81 were substituted by the artificially worn occlusal surfaces of specimen S23w and S81w, respectively.

Information for material properties such as the elastic modulus – E, and the Poisson’s ratio were collected from the literature [57]–[61] and summarized in Table 1. All the biological materials represented in the models were considered homogeneous, linearly elastic and isotropic, assumptions that are regularly applied with simpler continuum mechanics models [62]–[66].

**Table 1:** Elastic properties of dental and bone tissues.

Materials	E^b^ (GPa)	Poisson’s ratio	References
Enamel	84.1	0.3	57
Dentine	18.6	0.31	58
Pulp	0.002	0.45	59
PDL^a^	0.0689	0.45	60
Alveolar bone	11.5	0.3	61

a Periodontal ligament;

b elastic modulus.

Boundary constraints were applied to the medial and distal cut surfaces of the mandible section following indications provided by Benazzi et al. [56]: the medial nodes were restrained only in x-axis translation (linguo-buccally), while the distal nodes were restrained both in the y- and z-axes (supero-inferiorly and medio-distally, respectively). The load (uniform pressure) was distributed proportionally according to the occlusal contact areas detected in the OFA software (Figure 8) and was such that the magnitude of the resultant vector was equal to 100 N. A large range of occlusal loads have been proposed in the literature. Nonetheless, since we are interested in the patterns of stress distribution rather than predicting realistic loads that cause fractures of the tooth, the magnitude of the occlusal load is not a crucial factor, since at each material point of the model the stress is linearly proportional to the magnitude of force applied [56], [64].

The stress state patterns were qualitatively and quantitatively compared according to the first maximum principal stresses criterion for brittle materials [29], [30], [56], [65], [66], wherein the stresses inform about tensile behaviour in specific sites of the volumetric meshes.

## Supporting Information

Figure S1 **Basic steps to create a volumetric mesh for specimen S126 (lower right second premolar - RP2).** A and B show dental tissues and supporting structures after segmentation; PDL = periodontal ligament. C, the FE mesh consisting of 840,455 10-noded tetrahedral elements. B = buccal; D = distal; L = lingual; M = mesial.
(TIF)

Figure S2 **Cast of specimen S23 mounted in the dental articulator (PROTAR, KaVo Dental GmbH).** In order to perform artificial attrition, setup of the articulator condyle boxes derived from the individual occlusal movements extracted from the macrowear on the crowns, following Kullmer et al. [22]. A, frontal view. B, right lateral view.
(TIF)

Table S1 Numbers of nodes and tetrahedral elements for each specimen.
(DOC)

Video S1 **Simulation of the individual occlusal “power stroke” of specimen S23 applying the Occlusal Fingerprint Analyser (OFA) software.** The OFA calculates a relief-guided pathway of antagonistic tooth rows from collision detection, deflection and break-free algorithms for user-defined timesteps. The contact areas of maximum intercuspation have been chosen for applying loads in the FE models.
(MP4)

Video S2 **Simulation of the individual occlusal “power stroke” of specimen S81 applying the Occlusal Fingerprint Analyser (OFA) software.** The OFA calculates a relief-guided pathway of antagonistic tooth rows from collision detection, deflection and break-free algorithms for user-defined timesteps. The contact areas of maximum intercuspation have been chosen for applying loads in the FE models.
(MP4)

Video S3 **Simulation of the individual occlusal “power stroke” of specimen S5 applying the Occlusal Fingerprint Analyser (OFA) software.** The OFA calculates a relief-guided pathway of antagonistic tooth rows from collision detection, deflection and break-free algorithms for user-defined timesteps. The contact areas of maximum intercuspation have been chosen for applying loads in the FE models.
(MP4)

Video S4 **Simulation of the individual occlusal “power stroke” of specimen S126 applying the Occlusal Fingerprint Analyser (OFA) software.** The OFA calculates a relief-guided pathway of antagonistic tooth rows from collision detection, deflection and break-free algorithms for user-defined timesteps. The contact areas of maximum intercuspation have been chosen for applying loads in the FE models.
(MP4)

Video S5 **Simulation of the individual occlusal “power stroke” of specimen S23w applying the Occlusal Fingerprint Analyser (OFA) software.** The OFA calculates a relief-guided pathway of antagonistic tooth rows from collision detection, deflection and break-free algorithms for user-defined timesteps. The contact areas of maximum intercuspation have been chosen for applying loads in the FE models.
(MP4)

Video S6 **Simulation of the individual occlusal “power stroke” of specimen S81w applying the Occlusal Fingerprint Analyser (OFA) software.** The OFA calculates a relief-guided pathway of antagonistic tooth rows from collision detection, deflection and break-free algorithms for user-defined timesteps. The contact areas of maximum intercuspation have been chosen for applying loads in the FE models.
(MP4)
