# Supplementary figures and images for: The Evolutionary Paradox of Tooth Wear: Simply Destruction or Inevitable Adaptation?
Source: PLoS One. 2013 Apr 24;8(4):e62263. doi: 10.1371/journal.pone.0062263 (PMC3634733; doi:10.1371/journal.pone.0062263)

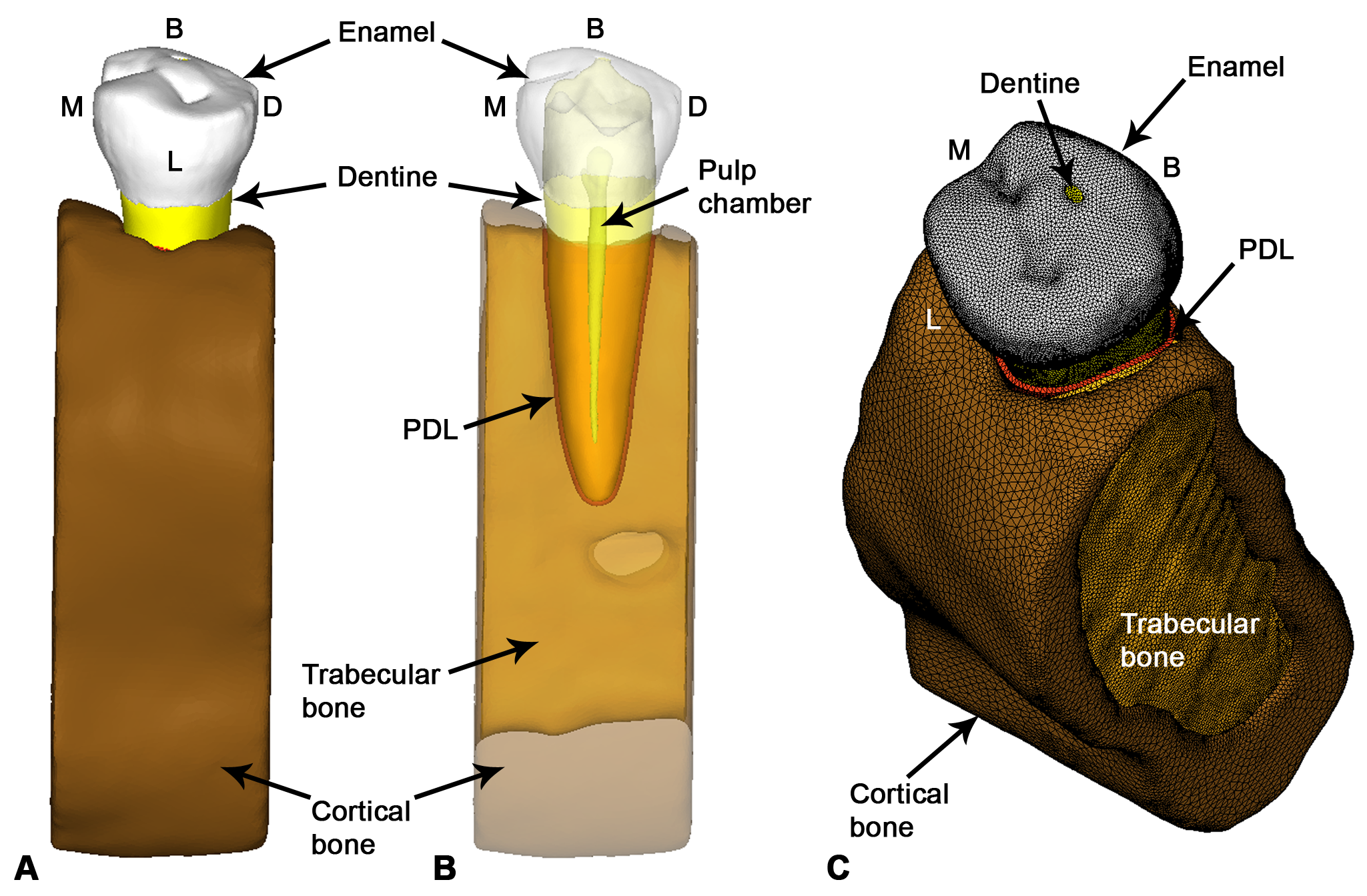

Supplement: Figure S1 — Basic steps to create a volumetric mesh for specimen S126 (lower right second premolar - RP2). A and B show dental tissues and supporting structures after segmentation; PDL = periodontal ligament. C, the FE mesh consisting of 840,455 10-noded tetrahedral elements. B = buccal; D = distal; L = lingual; M = mesial. (TIF) [file pone.0062263.s001.tif]

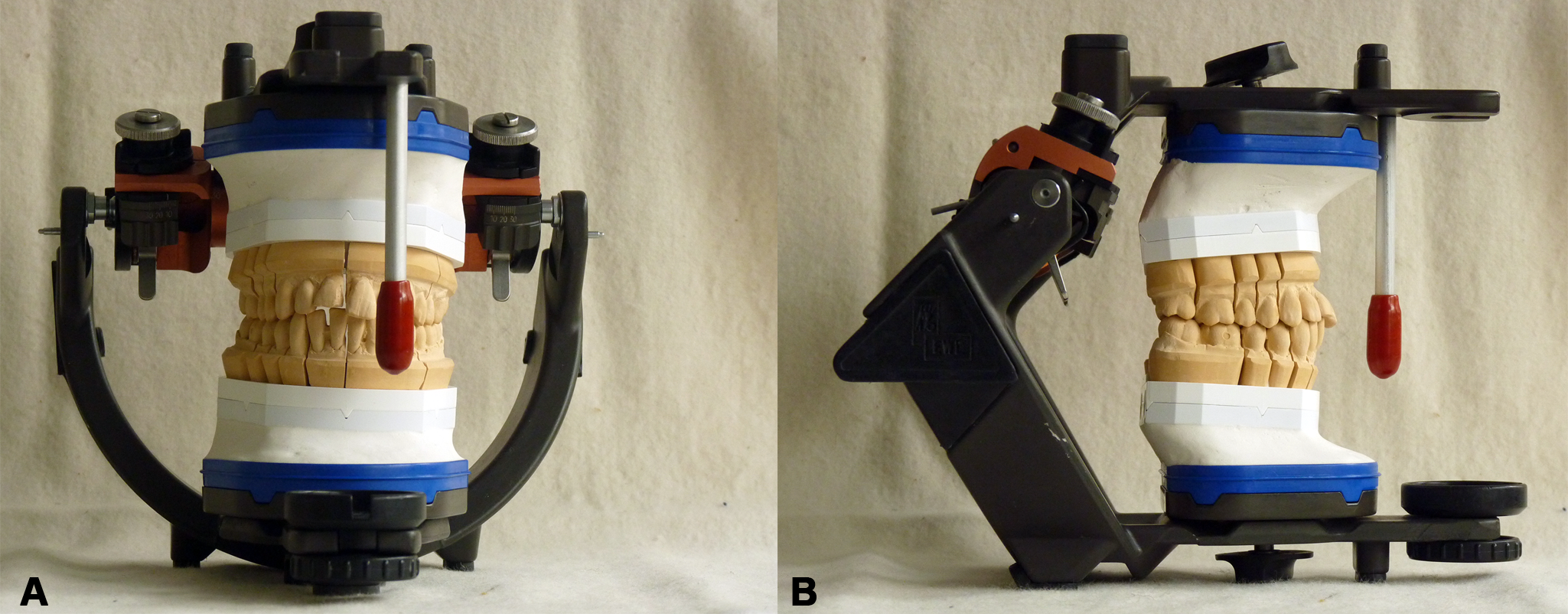

Supplement: Figure S2 — Cast of specimen S23 mounted in the dental articulator (PROTAR, KaVo Dental GmbH). In order to perform artificial attrition, setup of the articulator condyle boxes derived from the individual occlusal movements extracted from the macrowear on the crowns, following Kullmer et al. [22]. A, frontal view. B, right lateral view. (TIF) [file pone.0062263.s002.tif]
