# Supplementary material for: The Evolutionary Paradox of Tooth Wear: Simply Destruction or Inevitable Adaptation?
Source: PLoS One. 2013 Apr 24;8(4):e62263. doi: 10.1371/journal.pone.0062263 (PMC3634733; doi:10.1371/journal.pone.0062263)
Supplement: Table S1 — Numbers of nodes and tetrahedral elements for each specimen. (DOC) [file pone.0062263.s003.doc]

| **Table S1.** Numbers of nodes and tetrahedral elements for each specimen | | |
| --- | --- | --- |
|
| Specimen | Nodes | Tetrahedral elements |
| S5 | 1,640,392 | 1,169,079 |
| S23 | 1,095,672 | 774,819 |
| S81 | 1,177,621 | 836,631 |
| S126 | 1,197,767 | 840,455 |
| S23w^a^ | 1,123,709 | 793,701 |
| S81w^a^ | 1,212,938 | 860,774 |
| ^a^Artificially worn | | |
